# Lion’s Mane (*Hericium erinaceus*) Exerts Anxiolytic Effects in the rTg4510 Tau Mouse Model

**DOI:** 10.3390/bs12070235

**Published:** 2022-07-15

**Authors:** Mya N. Rodriguez, Stephen L. P. Lippi

**Affiliations:** Department Psychology, Angelo State University, San Angelo, TX 76909, USA; mrodriguez148@angelo.edu

**Keywords:** Alzheimer’s disease, anxiety, tau, mice, behavior, mushrooms

## Abstract

Alzheimer’s disease (AD) significantly impairs the life of an individual both cognitively and behaviorally. Tau and beta-amyloid (Aβ) proteins are major contributors to the etiology of AD. This study used mice modeling AD through the presence of tau pathology to assess the effects of *Hericium erinaceus* (*H. erinaceus*), also known as Lion’s mane, on cognitive and non-cognitive behaviors. Despite neurocognitive and neurobiological effects of *H. erinaceus* being seen in both healthy and transgenic mice, no research to date has explored its effects on mice with solely tau pathology. In this study, mice were placed on a diet supplemented with *H. erinaceus* or a standard rodent diet for 4.5 months in order to determine the effect of this medicinal mushroom on behavior. Tau mice given *H. erinaceus* had significantly shorter latencies to enter the center of the open field (OF) (*p* < 0.05) and spent significantly more time in the open arms of the elevated zero maze (EZM) (*p* < 0.001) compared to tau control mice. Mice given *H. erinaceus* spent significantly more time in the open arms of and made more head dips in the elevated zero maze (EZM) (*p* < 0.05). While *H. erinaceus* had anxiolytic effects, no improvements were seen in spatial memory or activities of daily living. These findings provide additional support for the anxiolytic effects of *H. erinaceus* and point to its potential benefit as a therapeutic for anxiety in AD.

## 1. Introduction

Alzheimer’s disease (AD) is a neurodegenerative disorder that negatively affects memory, language, problem-solving, consciousness, reasoning, and everyday living. AD is characterized by the unchecked accumulation of two proteins: the microtubule-associated protein tau and beta-amyloid (Aβ). β-amyloid clumps together and forms plaques in extracellular spaces between neurons, resulting in synapse loss [1]. Tau proteins normally support a healthy neuron by stabilizing microtubules [2]. Neurofibrillary tangles, or tau tangles, begin to form from these tau proteins after they have become hyperphosphorylated and dissociate from microtubules [3]; this leads to destabilization of the microtubule network and impairs neuronal communication [4]. In the AD brain, amyloid plaques and tau tangles are strongly associated with the development and progression of AD.

Treatments for AD only provide some improvements in the life of the individual that may slow the progression of the disease but do not terminate it. Investigation into treatments that may play a potential preventative role are warranted, since no new pharmacological treatments have been approved for AD since 2003 [5,6]. There is an urgent need for treatment to become available; however, the lack of understanding of AD, timely clinical trials, and expensive research [7] have led to the high failure rate of candidate disease-modifying treatments (DMTs) [8]. Lifestyle factors, such as diet, are more easily managed and implemented into a daily routine by individuals compared to pharmacological treatments. Mushrooms are easily accessible and do not have adverse side effects shown by prescription drugs [5]. The medicinal mushroom, *Hericium erinaceus* (*H. erinaceus*), has been shown to have numerous beneficial neurocognitive effects which may play a protective role against the development of AD.

*H. erinaceus* is an edible mushroom that is highly valued due to its various health benefits [9]. This medicinal mushroom has been shown to have many neurocognitive and mental benefits, and leads to improvements in the immune system due to its constituents hericenones and erinacines [5,10,11]. Hericenones (aromatic compounds with molecular weights between 330.4 and 598.9 g/mol [12]) and erinacines (diterpenoids with molecular weights between 360.5 and 506.6 g/mol [12]) are compounds that can be isolated from the fruiting bodies of mushrooms and cross the blood brain barrier (BBB) [13,14]. Their ability to cross the BBB is what allows *H. erinaceus* to have a high therapeutic potential. Additional benefits of *H. erinaceus* include the promotion of nerve growth factor (NGF) [15], promotion of brain-derived neurotrophic factor (BDNF), improvement of cognitive function [11], promotion of anti-inflammation, reduction in astrocyte activation, hippocampal neurogenesis, glial cell activation [16], reduction in nitric oxide (NO) production in BV2 microglia, and improvements in AD-related behaviors such as burrowing and nesting in mice [9] which can be viewed as analogous to activities of daily living in humans. *H. erinaceus* has also been shown to lessen anxiety and depression after four weeks of consumption in human subjects [17]. This is important since the onset of AD results in non-cognitive neuropsychiatric symptoms such as anxiety and depression which negatively affect the quality of life of the individual living with AD and their caregivers [18]. Thus, *H. erinaceus* possesses many beneficial qualities that may help combat dementia and AD [15].

Brandalise and colleagues (2017) [11] assessed the effects of *H. erinaceus* in wild-type (WT) mice. This was the first study to highlight the beneficial properties of *H. erinaceus* on healthy non-transgenic mice. One-month-old mice were administered a diet with 5% “Micotherapy Hericium” (0.025 g/g body weight) for two months. Researchers found both behavioral and biochemical effects of *H. erinaceus* administration. Mice receiving *H. erinaceus* had improved recognition memory and exploratory behavior in the novel object recognition task; this increased exploratory behavior toward novel objects was indicative of lower levels of anxiety [11]. Electrophysiological results also showed decreases in the stimulation failure rate of mossy fiber-CA3 neurons in the hippocampus when creating excitatory currents; these neurons are targeted in the neural pathway involving the perirhinal cortex, lateral entorhinal cortex, and dentate gyrus when the mice encounter novel objects [11]. This research supports the notion that *H. erinaceus* has beneficial effects on cognition and brain regions implicated in AD.

Researchers have also found that *H. erinaceus* can reduce Aβ plaque formation in the APPswe/PS1dE9 mouse model receiving erinacine A-enriched *H. erinaceus* Mycelia (HE-My) for 30 days [16]. Erinacines have been shown to stimulate the production of nerve growth factor (NGF) and offer neuroprotective properties [5,19]. In a series of biochemical assessments, HE-My administration presented multiple benefits, including: a reduction in the non-compact structure of plaques (the soluble form of Aβ plaques), an increase in insulin-degrading enzyme (IDE—an Aβ-degrading enzyme) in the cortex, and an increase in hippocampal neurogenesis. Tsai-Teng and colleagues (2016) [16] also found that continued administration of HE-My in mice from 30 days until 81 days improved burrowing and nesting behaviors in Activities of Daily Living (ADL) assessments.

*H. erinaceus* has also been shown to have beneficial effects on human subjects with mild AD. Li et al. (2020) [5] conducted a 1-year human pilot study with 49 eligible participants who consumed 350 mg capsules with 5 mg/g of erinacine A three times a day. Researchers found improvements in the blood biomarkers calcium, albumin, hemoglobin (Hb), superoxide dismutase (SOD), BDNF, and homocysteine (Hcy) [5]. Participants consuming the *H. erinaceus* mycelium capsules also exhibited improvements in APOE4, alpha-ACT (α-ACT), reductions in β-amyloid, and significant improvements in the Mini-Mental State Examination (MMSE) and Instrumental Activities of Daily Living (IADL), representing improved cognition and higher levels of independence [5]. Additionally, *H. erinaceus* has been shown to reduce anxiety and depression after four weeks in healthy female human participants [17]. These participants consumed *H. erinaceus* cookies containing 0.5 g of powdered fruiting body from the mushroom four times a day at any time of the day [17]. Researchers saw improvements as a result of *H. erinaceus* with the use of two scales: the Indefinite Complaints Index (ICI), an anxiety measure and a common scale used to measure the clinical effects of treatments, and the Center for Epidemiologic Studies Depression Scale (CES-D), a scale used to measure symptoms of depression by a self-report method [17]. Given the positive effects *H. erinaceus* has had on both healthy and cognitively impaired individuals, more research is warranted to continue exploring these benefits.

Previous studies have only assessed *H. erinaceus* supplementation in amyloid mouse models rather than tau [15,16,20]. As tau is a major constituent of AD pathology along Aβ plaques, it is important to assess the effects of *H. erinaceus* on a mouse model solely containing tau.

In the present study, the rTg4510 tau mouse model [21] was used to study the effects of *H. erinaceus* administration on cognitive and non-cognitive behaviors. This specific mouse model contains the P301L tau mutation; tau expression is driven in the forebrain as a result of the CamKIIa promoter system [21]. These mice show cognitive and behavioral impairments by four months of age and over time, behavior, tau accumulation, and brain atrophy worsen. Other tau mouse models, such as the JNPL3 have the same P301L tau mutation; however, they result in motor abnormalities which can impact behavioral assessments [22]. As we were interested in assessing whether *H. erinaceus* administration could rescue behavioral deficits in mice containing tangle pathology, this model was selected. Four total groups were evaluated in this study: tau mice on/off *H. erinaceus* and WT mice on/off *H. erinaceus*. Mice on the mushroom diet were supplemented with *H. erinaceus* through wet food. Mice underwent behavioral tests assessing general locomotor activity, anxiety-like and risk-taking behavior, and spatial cognition and memory. Additionally, activities of daily living (ADL), including burrowing and nesting were measured. It was hypothesized that *H. erinaceus* would decrease anxiety-like behaviors, increase locomotor activity, decrease deficits in spatial memory, and improve performance in ADL measures.

## 2. Materials and Methods

All procedures were approved by the Angelo State University Institutional Animal Care and Use Committee (IACUC) (approved protocol #21-204) and were in accordance with the National Institutes of Health guide for the care and use of Laboratory animals.

### 2.1. Animals

Experimental mice were bred by pairing female Fgf14^Tg(tetO-MAPT*P301L)4510Kha^ (tau) mice and male Tg(Camk2a-tTA)1Mmay (CaMKIIa promoter) mice (The Jackson Laboratory, Bar Harbor, ME, USA) in 2F:1M breeding harems. Tail snips from offspring were collected between days 11 and 14 and sent to Transnetyx, Inc. (Cordova, TN, USA) to appropriately genotype each animal. Offspring were weaned between 21 and 28 days old. Mice were then housed in separate cages (Animal Care Systems, Centennial, CO, USA) based on their sex, diet condition, and genotype (Table 1).

### 2.2. Housing

Mice were housed in an Optimice® caging system (Animal Care Systems, Centennial, CO, USA); each cage contained sani-chip bedding and was equipped with a nylabone for mice to chew and a nesting square. No more than three mice were housed in any single cage. All mice were maintained on a 12-h light/dark cycle (08:00–20:00 lights on). Food and water were provided *ad libitum*.

### 2.3. Dietary Supplementation

At four weeks of age, mice in the mushroom dietary condition began to receive Lion’s Mane Mushroom Mycelium Powder that was mixed into wet food (Host Defense Mushrooms, Fungi Perfecti, LLC., Olympia, WA, USA)); this continued throughout the duration of the study for four months. Each cage in each of the groups was administered 150 g of wet food and 100 g of standard dry food pellets. Wet food was made by combining laboratory water and dry food, waiting for the dry pellets to soften, and mixing by hand. WT and tau mice supplemented with *H. erinaceus* received 1/3 teaspoon of powder containing 1000 mg of Lion’s Mane and approximately 550 mg of (*H. erinaceus*) mycelium polysaccharides mixed into the 150 g of wet food. WT and tau control mice received 150 g of wet food with no *H. erinaceus* powder. All mice were provided food *ad libitum* and weights of the wet food along with the dry food were taken every four days to measure average food consumption.

### 2.4. Measures and Behavioral Testing

#### 2.4.1. Body Weights and Food Consumption Weights

Body weights were collected throughout the duration of the experiment every eight days (after every second food weight collection) to assess weight gain as a result of maturation and dietary condition. Food weights were collected every four days to verify that mice were consuming a sufficient amount of wet food in accordance with their experimental condition, in comparison to the dry food provided *ad libitum*. Due to group housing of mice, the amount of wet food and dry food consumed was calculated by weighing the amount of wet food and dry food remaining in the cage after four days, subtracting that from the original amount (150 g for wet food; 100 g for dry food), and dividing that total by the number of mice in the cage to yield an average.

#### 2.4.2. Open Field Test (OFT)

The Open Field Test (OFT) consists of a square enclosure that is used to assess general locomotor activity in rodents, exploratory behavior, mood, and anxiety [23]. The enclosure was a white plastic box that measures 45 × 45 × 40 cm. Mice were given a single five-minute trial while an overhead camera with SMART animal behavior software (Panlab, Harvard Apparatus, Holliston, MA, USA) measured the following variables: distance traveled in the center of the OFT (cm), percent time spent in the center, and latency (s) to first enter into the center of the box. The OFT box was cleaned with 70% ethanol between mice to eliminate olfactory cues. The number of fecal boli was also counted manually at the end of each trial before cleaning.

#### 2.4.3. Elevated Zero Maze (EZM)

The Elevated Zero Maze (EZM) is a behavioral test measuring anxiety-like behavior and approach/avoidance conflict in rodents [24]. It is elevated off the ground and consists of an elevated ‘0’ shaped platform with two enclosed arms and two open arms on opposite ends [25]. The mouse moves between the enclosed and open arms within a single five-minute trial. Mice were considered to be inside a given arm when all four paws were in that particular arm. Mice displaying higher levels of activity by exploring the open and exposed arms of the EZM are perceived as less anxious in comparison to those who spend more time in the enclosed and protected arms [24]. The following variables were measured: number of arm transitions, time spent in the open versus closed arms, and head dips assessing risk-taking behavior [26]. Between each mouse, the EZM apparatus was cleaned with 70% ethanol to eliminate olfactory cues. One mouse was removed from EZM data analysis due to falling off the maze.

#### 2.4.4. Morris Water Maze (MWM)

The Morris Water Maze (MWM) is a behavioral test of spatial memory where rodents must use visual cues to locate a clear platform hidden just below opaque water [27]. During a five-day acquisition period, mice were run through three trials per day. Visual cues were placed onto a curtain hanging outside of the tub. These cues aid the mice in learning the location of the hidden platform which remains stationary during the test. Mice were placed into the MWM facing the wall and were given 60 s to find the hidden platform. If the mouse did not find the platform in the allotted time, the mouse was guided towards the platform and placed there for 10 s. Each day, mice were run through a different sequence of three cues; the sequence was the same for each mouse. During each trial, the following variables were measured: percent time spent in the target quadrant, latency to reach the platform (s), thigmotaxicity (time (s) spent swimming along the border of the pool), and total distance swam (cm). An overhead camera connected to SMART animal behavior tracking software (Panlab, Harvard Apparatus, Holliston, MA, USA) recorded these variables. On day six, after the five 3-trial acquisition days, each mouse underwent a single probe trial where the platform was lowered for the animals to be unable to access it. This is meant to measure long-term memory. Mice began at a novel location (between two of the previous start locations). Target crosses when the platform was submerged, time spent in the target quadrant, and thigmotaxicity were measured on this probe day.

#### 2.4.5. Activities of Daily Living: Burrowing and Nesting

Activities of Daily Living (ADL) are common measures for studying noncognitive aspects of AD in rodents; these primarily include burrowing and nesting. These assess the tendency for mice to have normal activity levels and general animal welfare [28]. Burrowing was analyzed first. Burrowing was assessed by individually housing mice in a shoebox cage (Ancare, Bellmore, NY, USA) containing a PVC tube with one end closed, containing 250 g of pea-gravel (small rocks). The amount of removed pea gravel was measured after 2-h and the following morning. One mouse was removed from both ADL measures due to a water bottle spilling in the shoebox cage during the 2-h burrowing assessment, which could have potentially served as a stressor to the mouse and affected performance in the ADL measures.

Once the two burrowing measures were recorded, fresh sani-chip bedding was given to each cage along with 2.5 g of shredded white paper, which was sprinkled into the cage. The following morning, pictures were taken of each cage and its nest for scoring by a rater blind to condition. Two raters rated each nest on a scale of 1–5, with 1 being no nest was constructed and 5 being a complete nest was constructed [29]. After reliability in ratings was assessed, the scores were averaged and used for analysis.

### 2.5. Statistical Analysis

A 2 (genotype) × 2 (diet) × 2 (sex) × 5 (time) mixed ANOVA was run to assess changes in body weights overtime and a 2 (genotype) × 2 (diet) × 2 (sex) × 4 (time) mixed ANOVA was run to assess differences in the consumption of dry and wet food over the course of the experiment.

Dependent variables in the OFT, EZM, and ADL measures (burrowing and nesting) were assessed through 2 (genotype) × 2 (diet) × 2 (sex) factorial ANOVAs.

A 2 (genotype) × 2 (diet) × 2 (sex) × 5 (acquisition days of the MWM paradigm) mixed ANOVA was run for the following dependent variables in the MWM: percent time spent in the target quadrant, latency to reach the platform (s), total distance (cm) and thigmotaxicity. Target crosses on the final probe trial (when the platform was submerged), thigmotaxicity, and time spent in the target quadrant were measured with a 2 (genotype) × 2 (diet) × 2 (sex) factorial ANOVA.

Pairwise comparisons were assessed following significant main effects and simple effects analyses were run following any significant interactions with Bonferroni corrections. Greenhouse Geiser corrections were applied when sphericity was violated in mixed ANOVA analyses. *p* < 0.05 was considered statistically significant and *p* < 0.10 was considered trending. All analyses were run through SPSS v.26 (IBM Corp., Armonk, NY, USA) and graphs were made through GraphPad Prism (v.9.3.1) (San Diego, CA, USA).

## 3. Results

### 3.1. Animal Body Weights

**Figure 1:**
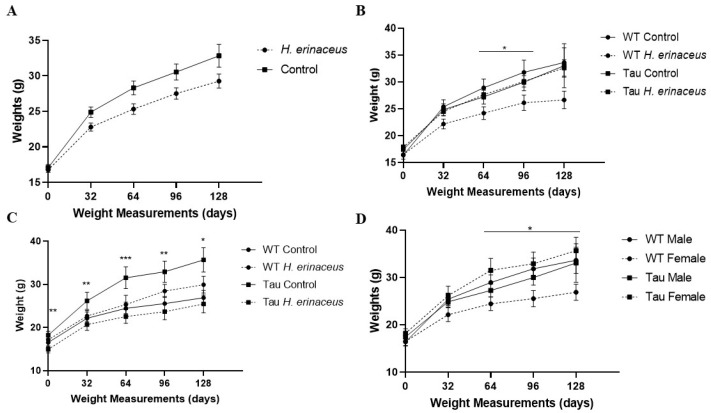
Weights over time. (**A**) Mice on the control diet (no *H. erinaceus*) weighed significantly more than those given *H. erinaceus*. (**B**) Male WT mice given the control diet weighed significantly more than their *H. erinaceus* counterparts at 64 days and 96 days; only male mice are graphed. (**C**) Female tau mice on the control diet weighed significantly more at each weighing point than female tau mice given *H. erinaceus*; only female mice are graphed. (**D**) Female tau mice weighed significantly more than female WT mice. Female tau mice weighed more than female WT mice at days 64, 96, and 128; only control diet mice are graphed. 0 = baseline; error bars represent mean ± SEM. (* *p* < 0.05, ** *p* < 0.01, *** *p* < 0.001).

There was a significant effect of day, *F*(1.679, 112.507) = 161.575, *p* < 0.001, η_p_^2^ = 0.707; as the experiment progressed, mice gained weight (Figure 1A). There was also a significant day × diet × sex × genotype interaction, *F*(1.679, 112.507) = 3.745, *p* < 0.05, η_p_^2^ = 0.053. In male WT mice, those given the control diet (no *H. erinaceus* in wet food) weighed significantly more than their mushroom counterparts only at 64 days (*p* < 0.05) and 96 days (*p* < 0.05) (Figure 1B). In female tau mice, those on the control diet weighed significantly more at each weighing time (baseline: *p* < 0.01; 32 days: *p* < 0.01; 64 days: *p* < 0.001; 96 days: *p* = 0.001; 128 days: *p* < 0.05) than those given *H. erinaceus* (Figure 1C). Finally, in control diet mice, female tau mice weighed significantly more than female WT mice at days 64 (*p* = 0.01), 96 (*p* < 0.05), and 128 (*p* < 0.05) (Figure 1D).

There was a significant between-subject effect of diet, *F*(1, 67) = 5.833, *p* < 0.05, η_p_^2^ = 0.08; control diet mice weighed more than those given *H. erinaceus* (Figure 1A). There was also a significant diet × sex × genotype interaction, *F*(1, 67) = 10.291, *p* = 0.002, η_p_^2^ = 0.133. In male mice, WT controls weighed significantly more than WT mushroom mice, *p* < 0.05 (Figure 1B). In female mice, tau mushroom mice weighed significantly less than tau control mice (*p* = 0.001) (Figure 1C). In control mice (no *H. erinaceus*), female tau mice weighed significantly more than female WT mice, *p* < 0.05 (Figure 1D).

### 3.2. Wet Food Consumption

**Figure 2:**
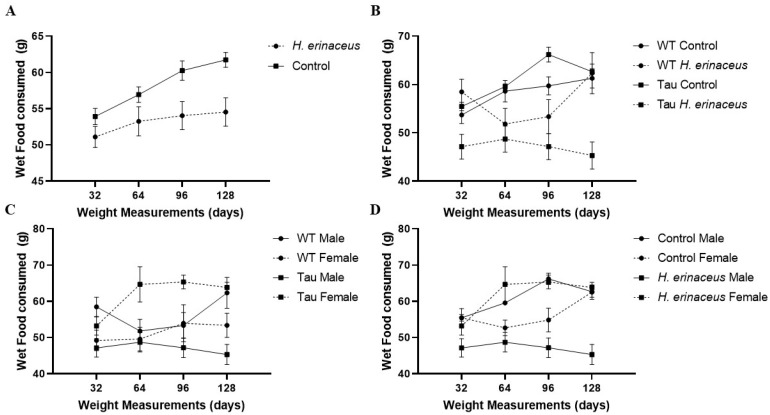
Wet food consumption over time. (**A**) Mice on the control diet (no *H. erinaceus*) ate more food than those given *H. erinaceus*. (**B**) Male control diet mice ate more wet food at day 64 (*p* < 0.01), 96 (*p* < 0.001), and 128 (*p* < 0.01) than male mushroom mice. Tau control mice ate significantly more wet food than tau mushroom mice; only male mice graphed. (**C**) Female mice ate more than male mice at 64 (*p* < 0.05) and 96 (*p* < 0.01) days. Female tau mice ate significantly more than male tau mice, *p* < 0.001. Tau female mice ate significantly more wet food than WT female mice (*p* = 0.001). WT male mice ate significantly more wet food than Tau male mice, *p* = 0.001; only *H. erinaceus* diet condition graphed. (**D**) Tau control mice ate more than tau mushroom mice at day 32 (*p* < 0.05) and 128 (*p* < 0.01). Female mice ate significantly more than male mice, *p* < 0.05. Male control mice ate significantly more wet food than male mushroom mice, *p* < 0.001. Female mushroom mice ate significantly more wet food than male mushroom mice, *p* < 0.001; only tau mice graphed. Error bars represent mean ± SEM.

There was a significant effect of time, *F*(2.689, 180.133) = 11.203, *p* < 0.001, η_p_^2^ = 0.143, indicating that mice ate more wet food over the course of the experiment (Figure 2A). There were also significant time × diet × sex, *F*(2.689, 180.133) = 4.511, *p* < 0.01, η_p_^2^ = 0.063, and time × diet × genotype, *F*(2.689, 180.133) = 3.831, *p* < 0.05, η_p_^2^ = 0.054 interactions. Male control diet mice ate more wet food at 64 (*p* < 0.01), 96 (*p* < 0.001), and 128 (*p* < 0.01) days than male mushroom mice (Figure 2B). Female mice given *H. erinaceus* ate more than male mice given *H. erinaceus* at 64 (*p* < 0.05) and 96 (*p* < 0.01) days (Figure 2C). Tau mice given the control diet ate more than tau mice given *H. erinaceus* at 32 (*p* < 0.05) and 128 (*p* < 0.01) days (Figure 2D).

There were significant effects of diet, *F*(1, 67) = 6.251, *p* < 0.05, η_p_^2^ = 0.085, and diet × sex, *F*(1, 67) = 8.399, *p* < 0.01, η_p_^2^ = 0.111, genotype × sex, *F*(1, 67) = 9.179, *p* < 0.01, η_p_^2^ = 0.120, and diet × sex × genotype, *F*(1, 67) = 13.792, *p* < 0.001, η_p_^2^ = 0.171 interactions. Control mice ate significantly more wet food than those on the *H. erinaceus* diet, *p* < 0.05 (Figure 2A). Female mice given mushrooms ate significantly more than males given mushrooms, *p* < 0.05 (Figure 2C). Female tau mice ate significantly more than male tau mice, *p* < 0.05 and tau females ate significantly more wet food than WT females, *p* < 0.05. Male tau mice given the control diet ate significantly more than male tau mice given *H. erinaceus*, *p* < 0.001 (Figure 2B). Female tau mushroom mice ate significantly more than male tau mushroom mice, *p* < 0.001 (Figure 2C). Interestingly, wet food consumption in genotype was dependent on sex in the mushroom condition: tau female mushroom mice ate significantly more than WT female mushroom mice, *p* = 0.001 while WT male mushroom mice ate significantly more than tau male mushroom mice, *p* = 0.001 (Figure 2C).

### 3.3. Dry Food Consumption

**Figure 3:**
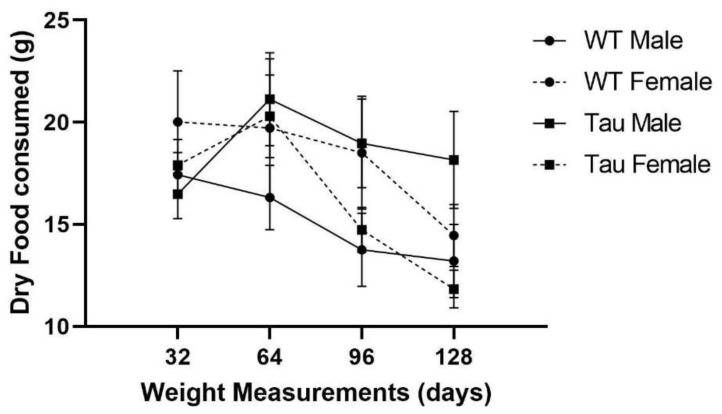
Dry food consumption over time. As the experiment progressed, mice ate less dry food, *p* < 0.01. Male tau mice consumed significantly more dry food than male WT mice (*p* < 0.05). Error bars represent mean ± SEM.

There was a significant effect of time, *F*(1.795, 120.248) = 8.314, *p* = 0.001, η_p_^2^ = 0.110; mice ate less dry food as the experiment progressed. There was a significant sex × genotype interaction, *F*(1, 67) = 4.497, *p* < 0.05, η_p_^2^ = 0.063. Male tau mice ate significantly more dry food than male WT mice (*p* < 0.05) (Figure 3).

### 3.4. Open Field Test

#### 3.4.1. Latency to Enter the Center

**Figure 4:**
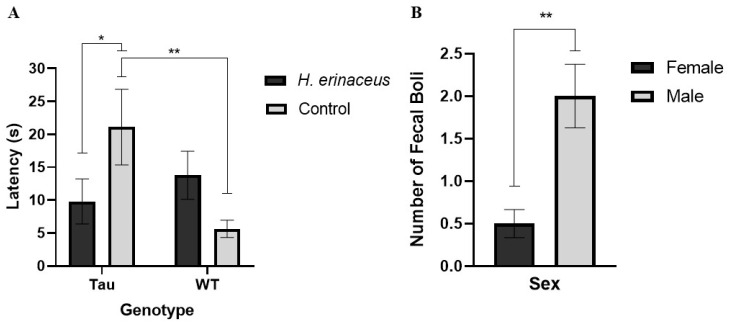
Open field test measures. (**A**) Tau control mice had significantly longer latencies to enter the center compared to WT control mice. Tau mice supplemented with *H. erinaceus* had significantly shorter latencies in entering the center of the OF compared to tau control mice. (**B**) Male mice defecated more than females. Error bars represent mean ± SEM (* *p* < 0.05, ** *p* < 0.01).

There was a significant diet × genotype interaction, *F*(1, 67) = 7.650, *p* < 0.01, η_p_^2^ = 0.102. Simple effects analysis revealed that tau control mice had significantly longer latencies to enter the center of the OF compared to tau mice given *H. erinaceus* (*p* < 0.05) and WT control mice (*p* < 0.01) (Figure 4A). Analysis also revealed a trending diet × sex interaction, *F*(1, 67) = 3.217, *p* = 0.077, η_p_^2^ = 0.046. Female mushroom mice (M = 8.630 s, SD = 11.875) took less time to enter the center of the OF than female control mice (M = 17.897 s, SD = 20.574). Male mushroom mice (M = 14.813 s, SD = 19.129) took longer to enter the center of the OF than male control mice (M = 10.216 s, SD = 16.930).

#### 3.4.2. Total Distance in the Center (cm)

There was a trending effect of diet, *F*(1, 67) = 2.873, *p* < 0.10, η_p_^2^ = 0.041. Mice in the mushroom diet (M = 1033.89 cm, SD = 1534.67) traveled a greater total distance in the center of the OFT compared to mice in the control group (M = 480.23 cm, SD = 522.65).

#### 3.4.3. Percent Time Spent in the Center 

There were no significant effects of diet, *F*(1, 67) = 2.464, *p* = 0.121, η_p_^2^ = 0.035, sex, *F*(1, 67) = 0.001, *p* = 0.979, η_p_^2^ = 0.000, or genotype, *F*(1, 67) = 0.085, *p* = 0.771, η_p_^2^ = 0.001 for percent time spent in the center of the OF.

#### 3.4.4. Fecal Boli

Analysis of fecal boli revealed a significant effect of sex, *F*(1, 67) = 10.227, *p* = 0.002, η_p_^2^ = 0.132. Males left significantly more fecal boli than females during their time in the OFT (*p* < 0.01) (Figure 4B). There were no significant effects of genotype, *F*(1, 67) = 0.049, *p* = 0.825. There were no differences between tau mice (M = 1.181, SD = 1.929) and WT mice (M = 1.283, SD = 2.162). Additionally, there were no significant effects of diet, *F*(1, 67) = 0.177, *p* = 0.734. There were no differences between mice administered *H. erinaceus* (M = 1.153, SD = 1.767) and mice consuming the control diet (M = 1.311, SD = 2.311). 

### 3.5. Elevated Zero Maze 

#### 3.5.1. Head Dips

**Figure 5:**
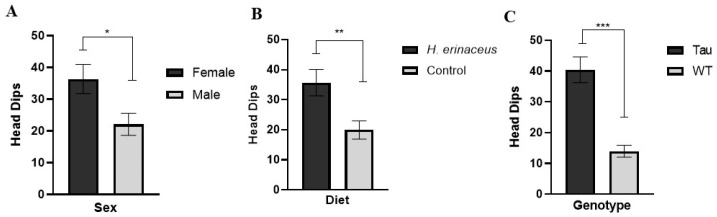
Head Dips in the EZM. (**A**) Female mice made significantly more head dips than male mice. (**B**) Mice given *H. erinaceus* made significantly more head dips than control mice. (**C**) Tau mice made significantly more head dips than WT mice. Error bars represent mean ± SEM (* *p* < 0.05, ** *p* = 0.01, *** *p* < 0.001).

There was a significant effect of sex, *F*(1, 66) = 6.377, *p* < 0.05, η_p_^2^ = 0.088, a significant effect of diet, *F*(1, 66) = 7.107, *p* = 0.010, η_p_^2^ = 0.097, and a significant effect of genotype, *F*(1, 66) = 29.143, *p* < 0.001, η_p_^2^ = 0.306. Female mice made significantly more head dips than male mice (*p* < 0.05) (Figure 5A). Mice given *H. erinaceus* made significantly more head dips than control mice (*p* = 0.010) (Figure 5B) and tau mice made significantly more head dips than WT mice (*p* < 0.001) (Figure 5C).

#### 3.5.2. Total Transitions 

There was a trending effect of sex, *F*(1, 66) = 3.108, *p* = 0.083, η_p_^2^ = 0.045. Female mice (M = 6.259, SD = 9.918) made more transitions compared to male mice (M = 3.127, SD = 4.993).

#### 3.5.3. Percent Time Spent in the Open Arms

**Figure 6:**
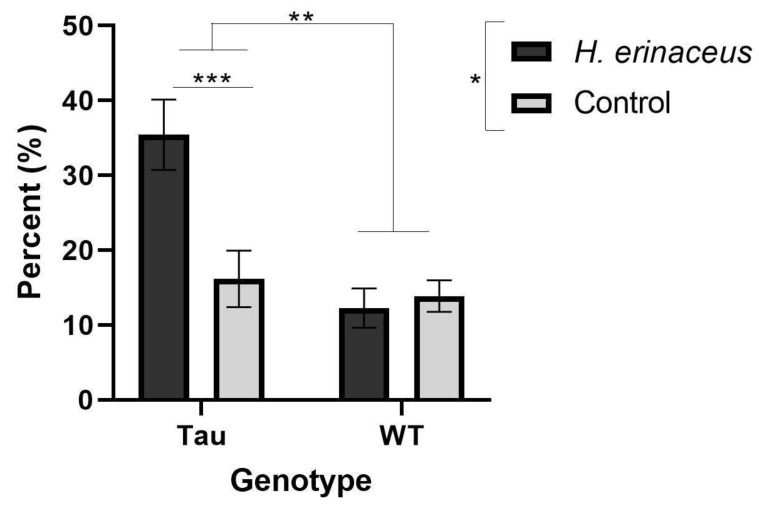
Percent time spent in the open arms of the EZM. Tau mice given *H. erinaceus* spent significantly more time in the open arms compared to tau control mice and tau mice spent more time in the open arms compared to WT mice. Mice given *H. erinaceus* spent more time in the open arms compared to control mice. Error bars represent mean ± SEM (* *p* < 0.05, ** *p* < 0.01, *** *p* = 0.001).

There was a significant effect of diet, *F*(1, 66) = 4.878, *p* < 0.05, η_p_^2^ = 0.069, a significant effect of genotype, *F*(1, 66) = 10.054, *p* = 0.002, η_p_^2^ = 0.132, and a significant diet × genotype interaction, *F*(1, 66) = 7.099, *p* = 0.010, η_p_^2^ = 0.097. Mice given *H. erinaceus* spent significantly more time in the open arms compared to control mice (*p* < 0.05). Tau mice spent significantly more time in the open arms compared to WT mice (*p* < 0.01) (Figure 6). Simple effects analysis revealed that tau mice given *H. erinaceus* spent significantly more time in the open arms compared to tau control mice (*p* = 0.001) (Figure 6).

### 3.6. Morris Water Maze 

#### 3.6.1. Latency to Platform 

**Figure 7:**
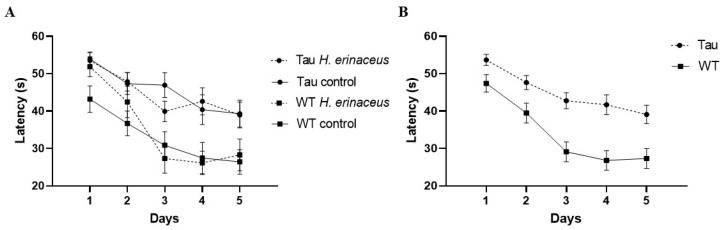
Latency to find the platform in the MWM. (**A**) As the days progressed, mice spent less time finding the platform (*p* < 0.001). (**B**) Tau mice had significantly longer latencies to find the platform compared to WT mice (*p* < 0.001) Error bars represent mean ± SEM.

There was a significant effect of day, *F*(4, 268) = 23.470, *p* < 0.001, η_p_^2^ = 0.259. As the days progressed, mice found the platform faster (Figure 7A). A between-subjects effect of genotype was also seen, *F*(1, 67) = 28.018, *p* < 0.001, η_p_^2^ = 0.295. Tau mice had significantly longer latencies to find the platform compared to WT mice, *p* < 0.001 (Figure 7B).

#### 3.6.2. Percent Time Spent in the Target Quadrant 

**Figure 8:**
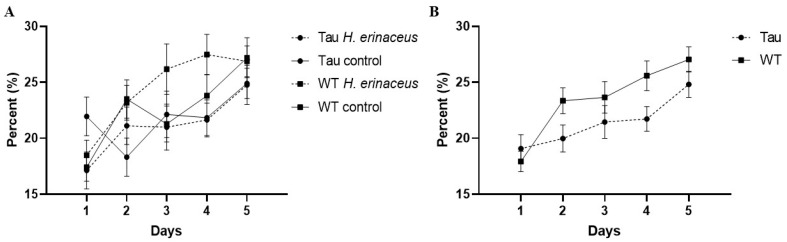
Percent time spent in the target quadrant of the MWM. (**A**) As the days progressed, mice spent more time in the target quadrant (*p* < 0.001). (**B**) WT mice significantly spent more time in the target quadrant compared to tau mice (*p* < 0.05). Error bars represent mean ± SEM.

There was a significant effect of day, *F*(3.444, 230.780) = 10.059, *p* < 0.001, η_p_^2^ = 0.131. As the days progressed, mice spent more time in the target quadrant (Figure 8A). There was also a significant day × diet × genotype × sex interaction, *F*(3.444, 230.780) = 2.567, *p* < 0.05, η_p_^2^ = 0.037. Simple effects analysis revealed that Tau female control mice spent significantly more time in the target quadrant compared to tau male control mice on day three (*p* < 0.05). A between-subjects effect of genotype was also seen, *F*(1, 67) = 4.926, *p* < 0.05, η_p_^2^ = 0.068. WT mice spent significantly more time in the target quadrant compared to tau mice (*p* = 0.03) (Figure 8B).

#### 3.6.3. Thigmotaxicity

**Figure 9:**
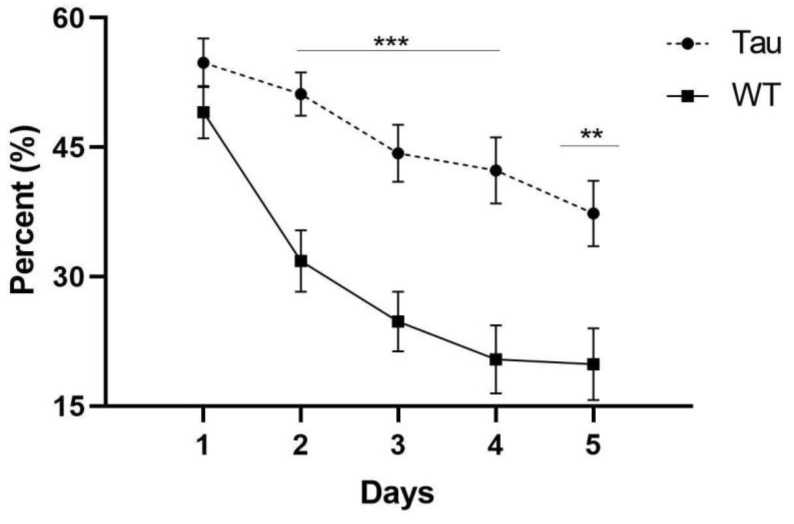
MWM thigmotaxicity by genotype. As the days progressed, tau mice spent significantly more time around the border of the MWM compared to WT mice. Tau mice spent significantly more time along the border on days 2–4 and 5 compared to WT mice. Error bars represent mean ± SEM. (** *p* < 0.01, *** *p* < 0.001).

There was a significant effect of day, *F*(3.024, 202.629) = 30.430, *p* < 0.001, η_p_^2^ = 0.312. As the days progressed, mice spent less time swimming along the border of the pool. A significant day × genotype interaction was also seen, *F*(3.024, 202.629) = 3.651, *p* = 0.013, η_p_^2^ = 0.052. Simple effects analysis revealed that WT mice spent significantly less time around the border than tau mice on days 2–4 (*p* < 0.001) and 5 (*p* < 0.01) (Figure 9). A between-subjects effect of genotype was also seen, *F*(1, 67) = 17.424, *p* < 0.001, η_p_^2^ = 0.206. Tau mice spent significantly more time along the border than WT mice (*p* < 0.001).

#### 3.6.4. Total Distance 

**Figure 10:**
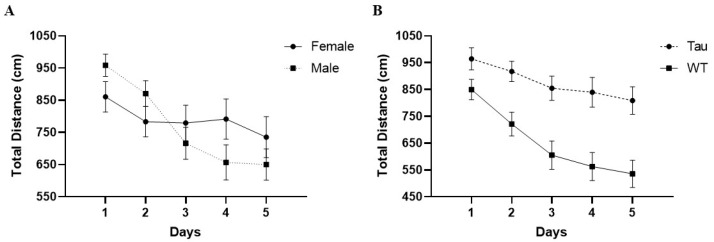
Total distance swam in the MWM. (**A**) As the days progressed, mice swam less total distance, with male mice specifically traveling shorter distances on days 3 through 5 compared to day 1 (*p* < 0.001). Female mice did not show significant differences in distances throughout the training days. (**B**) As the days progressed, tau mice traveled a significantly greater distance than WT mice (*p* < 0.001). Error bars represent mean ± SEM.

There was a significant effect of day, *F*(4, 268) = 9.995, *p* < 0.001, η_p_^2^ = 0.130. As the days progressed, mice swam shorter total distances. There was also a significant day × sex interaction, *F*(4, 268) = 2.724, *p* < 0.05, η_p_^2^ = 0.039. While females did not show a significant decrease in total distance swam across the acquisition days, male mice did. Males swam significantly less total distance on days 3, 4, and 5 compared to day 1 (*p* < 0.001) (Figure 10A). There was also a significant effect of genotype, *F*(1, 67) = 29.733, *p* < 0.001, η_p_^2^ = 0.307. Tau mice traveled a significantly greater total distance than WT mice (*p* < 0.001) (Figure 10B).

#### 3.6.5. Latency to Platform (Probe Day)

**Figure 11:**
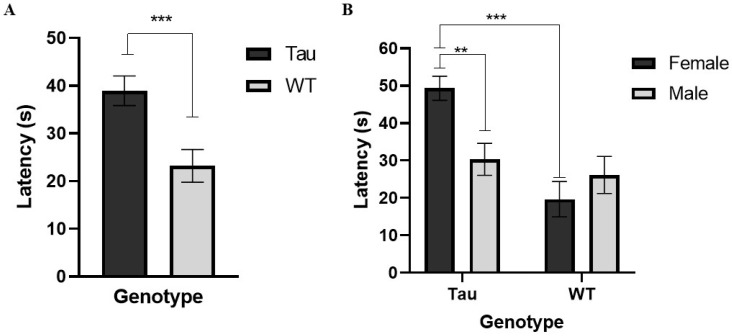
MWM probe trial latency to first cross platform. (**A**) Tau mice had a significantly longer latency to first reach where the platform would be than WT mice. (**B**) Female WT mice took significantly less time than female tau mice to first cross where the platform would be and female tau mice had a significantly longer latency to first cross where the platform would be than male tau mice. Error bars represent mean ± SEM (** *p* < 0.01, *** *p* ≤ 0.001).

There was a significant effect of genotype, *F*(1, 67) = 13.264, *p* = 0.001, η_p_^2^ = 0.165. Tau mice had a significantly longer latency to first reach where the platform would be than WT mice (*p* = 0.001) (Figure 11A). There was also a significant genotype × sex interaction, *F*(1, 67) = 7.911, *p* < 0.01, η_p_^2^ = 0.106. Female WT mice took significantly less time than female tau mice to first reach where the platform would be (*p* < 0.001). Additionally, female tau mice had a longer latency in first reaching where the platform would be than male tau mice (*p* < 0.01) (Figure 11B).

#### 3.6.6. Target Crossings (Probe Day)

**Figure 12:**
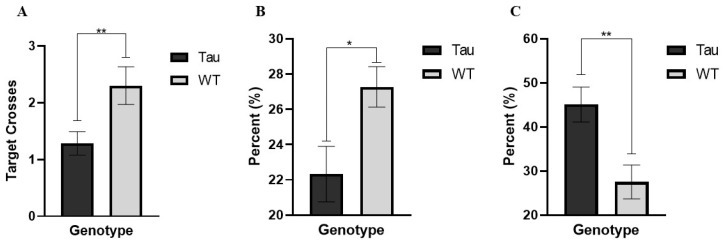
Additional MWM Probe Trial measures. (**A**) WT mice crossed the target significantly more than tau mice. (**B**) WT mice spent significantly more time in the target quadrant on the probe day than tau mice. (**C**) Tau mice spent significantly more time in the border (greater thigmotaxicity) than WT mice. Error bars represent mean ± SEM (* *p* < 0.05, ** *p* < 0.01).

There was a significant effect of genotype, *F*(1, 67) = 7.373, *p* < 0.01, η_p_^2^ = 0.099. WT mice made significantly more crosses over the target than tau mice (*p* < 0.01) (Figure 12A). There was also a significant genotype × sex interaction, *F*(1, 67) = 4.533, *p* < 0.05, η_p_^2^ = 0.063. Female WT mice made significantly more target crosses than female tau mice (*p* < 0.01).

#### 3.6.7. Percent Time Spent in the Target Quadrant (Probe Day)

Analysis revealed a significant effect of genotype, *F*(1, 67) = 5.267, *p* < 0.05, η_p_^2^ = 0.073. WT mice spent significantly more time in the target quadrant on the probe day than tau mice (*p* < 0.05) (Figure 12B). There was also a trending effect of diet, *F*(1, 67) = 3.616, *p* = 0.062, η_p_^2^ = 0.051. Mushroom mice (M = 23.054, SD = 9.64) spent less time in the target quadrant compared to control mice (M = 26.974, SD = 8.09).

#### 3.6.8. Thigmotaxicity (Probe Day)

There was a significant effect of genotype, *F*(1, 67) = 10.724, *p* = 0.002, η_p_^2^ = 0.138. Tau mice spent significantly more time in the border on the probe day compared to WT mice (*p* < 0.01) (Figure 12C).

### 3.7. Activities of Daily Living 

#### 3.7.1. Burrowing

**Figure 13:**
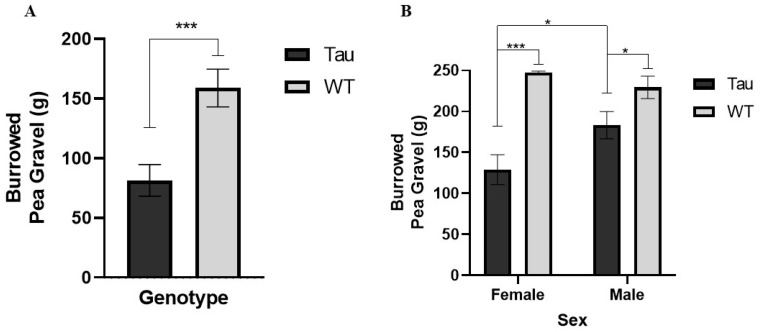
Burrowing assay measures. (**A**) Two-hour burrowing assessment. WT mice burrowed significantly more pea-gravel than tau mice after 2 h. (**B**) At the overnight measure, female WT mice burrowed significantly more than female tau mice. Male tau mice burrowed significantly more than female tau mice and male WT mice burrowed significantly more than male tau mice. Error bars represent mean ± SEM (* *p* < 0.05, *** *p* ≤ 0.001).

Analysis of pea-gravel burrowed after 2 h revealed a significant effect of genotype, *F*(1, 66) = 13.805, *p* < 0.001, η_p_^2^ = 0.173. WT mice burrowed significantly more pea-gravel than tau mice (*p* < 0.001) (Figure 13A). There was also a significant diet × genotype × sex interaction, *F*(1, 66) = 8.512, *p* < 0.01, η_p_^2^ = 0.114. Female WT mushroom mice burrowed significantly more than male WT mushroom mice (*p* < 0.05). Male tau mushroom mice burrowed significantly more than female tau mushroom mice (*p* = 0.020). Female WT mushroom mice burrowed more than female tau mushroom mice (*p* < 0.001). Male WT control mice burrowed significantly more than male tau control mice (*p* = 0.020).

Analysis of overnight pea-gravel burrowed revealed a significant effect of genotype, *F*(1, 66) = 25.586, *p* < 0.001, η_p_^2^ = 0.279, a significant genotype × sex interaction, *F*(1, 66) = 4.475, *p* = 0.038, η_p_^2^ = 0.063, and a significant diet × genotype × sex interaction, *F*(1, 66) = 4.626, *p* = 0.035, η_p_^2^ = 0.065. Male tau mice burrowed more than female tau mice (*p* < 0.05) (Figure 13B). Female WT mice burrowed more than female tau mice (*p* < 0.001). Male WT mice burrowed more than male tau mice (*p* < 0.05) (Figure 13B). Male tau mushroom mice burrowed significantly more than female tau mushroom mice (*p* < 0.001). Female tau control mice burrowed more than female tau mushroom mice (*p* = 0.013). Female WT mushroom mice burrowed more than female tau mushroom mice (*p* < 0.001).

#### 3.7.2. Nesting

**Figure 14:**
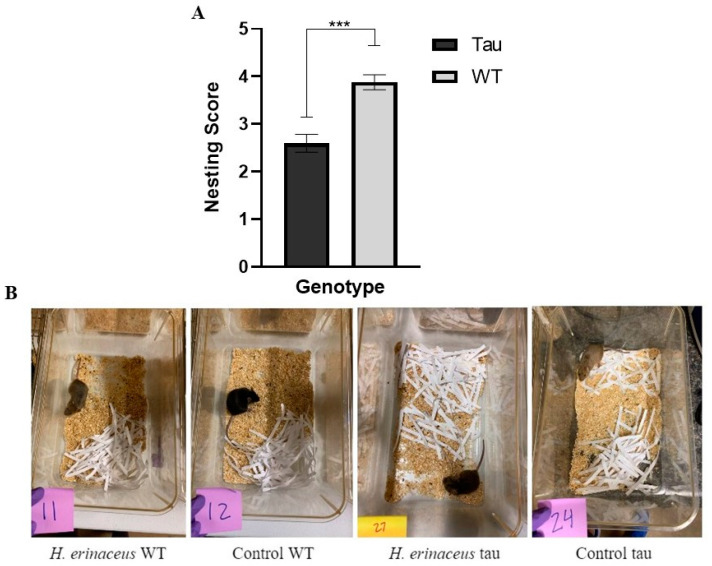
Nesting behavior. (**A**) WT mice built significantly better nests than tau mice. (**B**) Representative nest by mushroom condition and genotype; numbers represent randomly assigned IDs for raters. Error bars represent mean ± SEM (*** *p* < 0.001).

Nests were scored by two raters blind to experimental conditions. There was a strong agreement between the scores of both raters, α = 0.950; analysis was conducted on the resulting average nest score. There was a significant effect of genotype, *F*(1, 66) = 25.222, *p* < 0.001, η_p_^2^ = 0.276 (Figure 14A). WT mice built significantly better nests than tau mice (*p* < 0.001) (Figure 14B).

## 4. Discussion

This study sought to assess the effects of *H. erinaceus* on a tauopathy mouse model. We hypothesized that *H. erinaceus* would decrease anxiety-like behaviors, increase locomotor activity, decrease deficits in spatial memory, and improve performance in ADL measures. Overall, results indicate that *H. erinaceus* had significant anxiolytic effects and increased locomotor activity, in agreement with previous literature. However, *H. erinaceus* led to no improvements in spatial memory or activities of daily living.

Tau mice given *H. erinaceus* entered the center of the OFT apparatus faster than tau mice on the control diet, signaling decreased anxiety. Additionally, mice given *H. erinaceus* traveled a greater distance in the center of the OFT compared to control mice. These results are consistent with studies using WT mice and the OFT to assess the effects of *H. erinaceus* [30,31]. These studies showed that mice consuming *H. erinaceus* spent more time in the center of the OFT. Increased locomotor activity was also seen in mice consuming *H. erinaceus;* tau *H. erinaceus* mice entered the center significantly faster than tau control mice and *H. erinaceus* mice traveled a greater average distance in the center. This finding of increased locomotion is consistent with recent literature which showed that WT mice consuming *H. erinaceus* had increased locomotor activity in the Y maze [32] and longer exploration times in the emergence test, a variant assessment to the OFT [11].

Defecation is also a variable associated with emotionality, specifically stress and anxiety [33,34,35]. In the current study, males defecated significantly more than females, indicating higher levels of anxiety in males. This is a common finding consistent with past and current literature [33,36,37,38]. In addition to defecation denoting stress responses, it has also been suggested that males defecate more than females as a “territory marking response” [33,36].

In the EZM, female mice made more head dips and total transitions than male mice. This is consistent with literature that has shown that females are typically more active than males in the EZM and elevated plus maze (EPM), a similar test to the EZM used to measure anxiety-like behaviors [24,39]. Head dips are typically indicative of risk-taking behavior and decreased anxiety [40], meaning that in this assessment females presented increased risk-taking behaviors than males. Overall, mice given *H. erinaceus* spent more time in the open arms than control mice. More importantly, tau mice given *H. erinaceus* spent the most time in the open arms of the EZM and made more head dips than tau control mice. This is an important finding, showing that *H. erinaceus* has anxiolytic effects in the rTg4510 tau mouse model. A recent study [30] also indicated that WT mice supplemented with *H. erinaceus* spent more time in the open arms of the EPM than control mice. Additionally, it has been shown that *H. erinaceus* significantly increased entries into the open arm and time spent in the open arm of the EPM in WT mice [41].

Results do not reveal significant effects of *H. erinaceus* during the acquisition days of the MWM. However, mice did learn as the days progressed, which is consistent with literature using the MWM and transgenic mice of AD [42,43] and studies specifically assessing the effects of *H. erinaceus* on spatial memory with the MWM on AD rodent models [20,44]. Past literature has consistently shown spatial memory impairments in rTg4510 mice during the MWM assessment at 2.5, 3, and 5.5 months [21,45], and the Barnes Maze [46], with tau mice performing worse than their non-transgenic counterparts. It is also important to note that *H. erinaceus* has shown no effects on spatial memory in WT mice [32], which is consistent with the effects on the transgenic and non-transgenic mice used in this study. More research is warranted on assessing the effects of *H. erinaceus* on spatial memory in both WT and AD mouse models. Past research implying improvements on memory as an effect of the mushroom have done this with the Novel Object Recognition (NOR) task [11,15] which is used as a measure for short term memory rather than MWM which typically measures long term memory.

There were no significant effects of *H. erinaceus* on the probe day of the MWM; only significant effects of genotype were seen. This is indicative of the dietary condition having no effect on long-term memory. This contradicts previous studies assessing the effects of *H. erinaceus* in AD models, which have shown mice that consuming *H. erinaceus* performed better than mice not consuming *H. erinaceus* on the probe day [20,47]. As this is the first study assessing the effects of *H. erinaceus* on spatial memory in tau mice, it can help fill the gap and push further assessment of spatial memory in this mouse model by *H. erinaceus* supplementation.

*H. erinaceus* did not lead to improvements in ADL measures; only significant effects of genotype were noted. WT mice burrowed significantly more pea-gravel and built significantly better nests than tau mice. Interactions between diet, genotype, and sex were also seen. These results reveal that female WT mushroom mice burrowed significantly more than male WT mushroom mice, and that female tau mushroom mice burrowed significantly less than male tau mushroom mice in the 2-h burrowing measure. Despite no main effects of diet being seen, it is still worth reporting as not many studies assessing the effects of *H. erinaceus* have investigated ADL measures. Tsai-Teng et al. (2016) [16] conducted a nesting assessment with APP/PS1 mice consuming *H. erinaceus*. Results showed that AD mice presented more deficits in nesting activities compared to their WT counterparts; a result consistent with this study. However, in contrast with the results of the current study, researchers found that the administration of *H. erinaceus* was able to alleviate deficits in APP/PS1 mice in the nesting assessment [16].

Noncognitive assessments, such as ADL measures, are important to include as they can help determine non-cognitive deterioration in AD. More importantly, previous literature has shown that *H. erinaceus* improves non-cognitive deficits in humans consuming the mushroom in capsules 3 times a day for 49 weeks through the Instrumental Activities of Daily Living Scale (IADL) [5]. The IADL is a test of independent living skills in humans, a construct that is parallel to the ADL measure in rodent models.

Animal weights and wet food consumption increased over the course of the experiment. Tau and WT control mice weighed more than tau and WT mice given *H. erinaceus*. Throughout this experiment, tau and WT control mice consumed more wet food than tau and WT mice given *H. erinaceus*. It has been previously shown that tau mice consume more food but weigh less [45]. However, in the current study, tau mushroom mice ate less than tau control mice, indicating that not all tau mice ate more wet food. Additionally, tau female control mice weighed significantly more than WT female control mice, a finding inconsistent with previous research [48]. Female WT mice consuming *H. erinaceus* weighed significantly more than female tau mice consuming *H. erinaceus* although tau female mice ate more wet food than WT female mice. This, again, may be due to the progression of the disease in the tau mouse model as suggested by past literature [48]. Male tau mice supplemented with *H. erinaceus* weighed significantly more than male WT mice consuming *H. erinaceus* although male mice given *H. erinaceus* ate less throughout the experiment. Ryu et al. (2018) [31] found that the *H. erinaceus* did not have effects on the natural weight gain of the mice. This could be due to differences perhaps in how the diet was given; Ryu et al. (2018) [31] administered *H. erinaceus* by oral gavage while mice in the current study received *H. erinaceus* as a powder mixed into wet food. Further research is warranted on the causes of these weight differences, as a result of *H. erinaceus* consumption as well as sex. Additionally, when studying the effects of dietary manipulation, including food consumption and mouse weight data can be advantageous for future researchers to consider.

The hericenones and erinacines in *H. erinaceus* may play a role in the neurocognitive benefits seen in this mushroom [11,15]. More specifically, researchers have found that erinacine A is the biggest contributor to these neurological benefits out of fifteen total erinacines (A–K, P, Q, R, S) [5,19]. Researchers have also found that hericenones C, D, and E contribute to the synthesis of NGF, while hericenone F can reduce inflammation [49]. Thus, these hericenones and erinacines may be responsible for the anxiolytic effects seen in this study and several others [11,17]. Additional research into these components of *H. erinaceus* is certainly warranted.

There have been multiple methods of administering *H. erinaceus* to WT mice. Ratto et al. (2019) [50] used 21.5-month-old male WT mice. The mushroom was administered as a drink mixed with He1 mycelium and sporophore, which were ethanol extracts able to be solubilized in water for the mixture. Mice drank this *ad libitum* for two months. Researchers found that *H. erinaceus* improved recognition memory in aging mice. Ryu et al. (2018) [31] used two-month-old male WT mice and administered either 20 or 60 mg/kg of *H. erinaceus* powder by oral gavage for four weeks. Mice received the powder by oral gavage four times a day. It was found that the mice receiving 60 mg/kg of *H. erinaceus* exhibited anxiolytic and antidepressant behaviors.

There have also been multiple methods of administering *H. erinaceus* to transgenic mice. Mori et al. (2011) [15] used five-week-old male ICR (Institute of Cancer Research) mice with injected amyloid peptides. Fruiting bodies of *H. erinaceus* were turned into powder and mixed with a standard powdered diet, allowing the concentration of *H. erinaceus* to be 5%. The mushroom was administered to mice *ad libitum* for 23 days. *H. erinaceus* helped to ameliorate memory deficits in mice by improving recognition memory in the NOR test but did not improve exploratory behavior or locomotor activity in the Y-maze assessment. Tzeng et al. (2018) [20] used five-month-old female APP/PS1 mice, isolating *H. erinaceus* mycelium erinacine A and *H. erinaceus* mycelium erinacine S to assess the effects of each. The mushroom was administered at 10 mg/kg and 30 mg/kg a day, respectively, to experimental condition, for 100 days by oral gavage. Results indicate that erinacine A recovered cognitive impairments in spatial memory and activities of daily living (burrowing and nesting) [20]. Tsai-Teng et al. (2016) [16] also used five-month-old female APP/PS1 mice. Researchers administered 300 mg/kg per day of the mushroom for 30 days by oral gavage, isolating *H. erinaceus* mycelium containing erinacine A and *H. erinaceus* ethanol extracts. Tsai-Teng et al. (2016) [16] found that *H. erinaceus* mycelium recovered behavioral deficits in transgenic mice during nesting assessments.

Discussing mushroom administration in past literature is important because numerous labs and researchers may not always use the same methods, and this may perhaps be why results differ. This is the first study to administer mushroom powder mixed into wet food *ad libitum* to mice, with a standard rodent diet also available for the mice to feed on *ad libitum*. The goal was to mimic voluntary dietary supplementation in humans and eliminate stress factors which might accompany administration through techniques such as oral gavage. In each study, different quantities of *H. erinaceus* mycelium and respective components of the mushroom were used. All studies showing behavioral improvements, including this study, used *H. erinaceus* mycelium. The results of this study indicate anxiolytic effects and increased locomotor activity, but no improvements in spatial memory and noncognitive behaviors. Thus, it is important to explore the effects of different doses, erinacines, and extracts on future models of AD, including those models solely expressing tau.

One limitation in this study was the administration of the wet food. Each cage was administered 150 g of wet food for the mice to eat *ad libitum*. Other studies assessing the effects of *H. erinaceus* on WT and AD mouse models have administered the mushroom by oral gavage [16,20,31]. However, we chose to allow mice to eat *ad libitum* to eliminate potential stress and more closely resemble humans’ voluntary consumption of food. Due to this type of administration, we were unable to calculate the exact amount of wet food consumed by each individual mouse. Instead, the amount of wet food consumed was calculated as an average (see Section 2.4.1). We also collected animals’ body weights throughout the course of the experiment every 8 days to assess maturation and whether food was being consumed.

Another limitation may be the rTg4510 mouse model itself. In this specific regulatable model, the P301L tau mutation and CaMKIIa promoter system result in progressive neurofibrillary tangle pathology within the forebrain and memory deficits over time, which can be lessened when given doxycycline [21,51]. However, as Gamache et al. (2019) [52] report, this phenotype may not be caused solely by the expressed tau. Gamache et al. (2019) [52] report that in this model, disruptions in genes, including fibroblast growth factor 14 (Fgf14) caused by insertion of the transgene itself may be responsible for the particular phenotypes reported. While this model still allows for researchers to assess tangle pathology and behavioral deficits as a function of age, when applying an intervention aimed at alleviating behavioral or biochemical deficits, researchers must consider other factors which may be causing the deficits in the first place, beyond simply the tau mutation.

This is the first study demonstrating that *H. erinaceus* has anxiolytic effects in the rTg4510 tau mouse model. Previous research has shown this in relation to amyloid and WT mouse models but has not demonstrated it in a strictly tau mouse model [11,16,50,53]. Many other studies have assessed the cognitive effects of *H. erinaceus* in other models of AD or cognitive decline/aging such as APP/swePS1dE9 [16,20], SAMP8 [54], ICR (Institute of Cancer Research) mice with injected amyloid peptides [15], and Sprague Dawley rats injected with d-galactose [44]. This is also one of the first studies to assess the effects of *H. erinaceus* on both female and male mice. Previous literature assessing the effects of *H. erinaceus* has only used either male [11,50,53] or female mice [16], and has not assessed both sexes, with the majority of studies primarily assessing the effects on male mice.

The effects of *H. erinaceus* on tau mouse models have not been as consistently analyzed as those in amyloid models. Because amyloid and tau both contribute to the development of AD, it is important to study both markers and note the behavioral differences found in each model. Research has shown that tau may be a stronger underlying factor in the development of AD than Aβ [55]. By studying the effects of *H. erinaceus* in this rTg4510 tau mouse model, we aim to help bridge the gap between results solely seen in amyloid models; these results provide new views on differences in behavior that can be seen when using amyloid vs. tau models.

Future biochemical assessments on the effects of *H. erinaceus* would be informative to help understand the mechanisms by which this mushroom improves behavior. Tsai-Teng et al. (2016) [16] assessed Insulin-degrading Enzyme (IDE), NGF, Glial Fibrillary Acidic Protein (GFAP), and APP through Western Blot analysis. Results showed that *H. erinaceus* increased IDE levels which ameliorated Aβ plaques, increased the ratio of NGF and proNGF, decreased levels of GFAP and, interestingly, did not affect levels of APP. Future research could use immunoblots to assess levels of tau species, SOD-1, GFAP, NGF, glucocorticoid, and BDNF in this tau mouse model. Analyzing these proteins could provide information about the mushroom’s effects on tau levels, oxidation, astrocyte activity and inflammation, fiber growth and survival, and maintenance of nerve cells. As the current study demonstrated anxiolytic effects of *H. erinaceus* in this tau mouse model, analyzing proteins such as glucocorticoid receptors could provide a more detailed connection between the brain and behavior.

## 5. Conclusions

Overall, this study demonstrated that supplementation of *H. erinaceus* through wet food for four months has anxiolytic effects on the rTg4510 tau mouse model but leads to no improvements in spatial memory nor activities of daily living. Although no improvements were found in spatial memory, the anxiolytic effects may serve a great benefit for caretakers or those living with AD seeking to add a supplement that can help lower anxiety.

## Figures and Tables

**Table 1:** Experimental Sample Sizes (Behavioral Analysis).

Diet	Tau	Wildtype (WT)	
*H. erinaceus*	**25** 12F, 13M	**16** 8F, 8M	**41** 20F, 21M
Control Diet	**17** 7F, 10M	**17** 7F, 10M	**34** 14F, 20M
			**N = 75**

## Data Availability

The data presented in this study are available upon request from the corresponding author, without undue reservation.
